# Comparative genomics of *Burkholderia multivorans*, a ubiquitous pathogen with a highly conserved genomic structure

**DOI:** 10.1371/journal.pone.0176191

**Published:** 2017-04-21

**Authors:** Charlotte Peeters, Vaughn S. Cooper, Philip J. Hatcher, Bart Verheyde, Aurélien Carlier, Peter Vandamme

**Affiliations:** 1 Laboratory of Microbiology, Ghent University, Ghent, Belgium; 2 Department of Microbiology and Molecular Genetics, University of Pittsburgh School of Medicine, Pittsburgh, PA, United States of America; 3 Department of Computer Science, University of New Hampshire, Durham, NH, United States of America; Institut National de la Recherche Agronomique, FRANCE

## Abstract

The natural environment serves as a reservoir of opportunistic pathogens. A well-established method for studying the epidemiology of such opportunists is multilocus sequence typing, which in many cases has defined strains predisposed to causing infection. *Burkholderia multivorans* is an important pathogen in people with cystic fibrosis (CF) and its epidemiology suggests that strains are acquired from non-human sources such as the natural environment. This raises the central question of whether the isolation source (CF or environment) or the multilocus sequence type (ST) of *B. multivorans* better predicts their genomic content and functionality. We identified four pairs of *B. multivorans* isolates, representing distinct STs and consisting of one CF and one environmental isolate each. All genomes were sequenced using the PacBio SMRT sequencing technology, which resulted in eight high-quality *B. multivorans* genome assemblies. The present study demonstrated that the genomic structure of the examined *B. multivorans* STs is highly conserved and that the *B. multivorans* genomic lineages are defined by their ST. Orthologous protein families were not uniformly distributed among chromosomes, with core orthologs being enriched on the primary chromosome and ST-specific orthologs being enriched on the second and third chromosome. The ST-specific orthologs were enriched in genes involved in defense mechanisms and secondary metabolism, corroborating the strain-specificity of these virulence characteristics. Finally, the same *B. multivorans* genomic lineages occur in both CF and environmental samples and on different continents, demonstrating their ubiquity and evolutionary persistence.

## Introduction

*Burkholderia cepacia* complex (Bcc) bacteria are rare but potentially virulent pathogens in cystic fibrosis (CF) patients [1]. Epidemiological surveys revealed that *B. multivorans* is the most prevalent Bcc CF pathogen in many countries [1–7]. The continued emergence of unique *B. multivorans* strains in CF patients suggests acquisition from non-human sources, such as the natural environment [8]. Environmental conditions or non-human hosts in which virulence factors might be adaptive can select for traits that confer virulence and natural environments could therefore serve as reservoirs of opportunistic pathogens [9, 10].

Multilocus sequence typing (MLST) is a well-established method for studying the epidemiology and population structure of Bcc organisms [11, 12]. The Bcc MLST scheme takes into account the allelic variation of seven housekeeping genes and each strain is defined by its unique allelic profile and multilocus sequence type (ST) [13]. Baldwin *et al.* [14] demonstrated that roughly one fifth of the clinical isolates in the Bcc PubMLST database had the same ST as environmental isolates, suggesting these isolates represent the same strain. A follow-up study demonstrated that several *B. multivorans* STs were globally distributed and that the natural environment (e.g. water and soil) may be an important reservoir for infection with this species [8].

*Burkholderia* genomes vary in size from 2.4 Mb (*Ca.* Burkholderia schumannianae UZHbot8) [15] to 11.5 Mb (*Burkholderia terrae* BS001) [16], are characterized by a high G+C content (62-68 mol%) and consist of multiple replicons [17, 18]. To gain insight into the overall genome biology of *B. multivorans*, we sequenced the genomes of eight isolates representing four distinct STs. For each ST, a CF and an environmental isolate were sequenced using the PacBio Single-Molecule Real-Time (SMRT) sequencing technology. The present study provides the first comprehensive comparative genome analysis of *B. multivorans* and assesses to which extent isolates with the same ST but from different origin (CF versus environmental) differ in genetic potential.

## Materials and methods

### Studied isolates

We searched the Bcc PubMLST database (http://pubmlst.org/bcc/) [13], identified four *B. multivorans* STs that included both CF and environmental (ENV) isolates and selected eight isolates for whole-genome sequencing (Table 1). Strains were grown aerobically on Tryptone Soya Agar (Oxoid) and incubated at 28°C. Cultures were preserved in MicroBank^™^ vials at -80°C.

**Table 1:** *B. multivorans* isolates included in the present study.

Isolate	Strain number	ST	Isolation source	Depositor
ST180-ENV	LMG 29305, J2943	180	Rhizosphere soil (United Kingdom, 2000)	J.R.W. Govan
ST180-CF	LMG 29313, 8335	180	CF sputum (Czech republic, 2011)	P. Drevinek
ST189-ENV	LMG 29309	189	Succulent soil (Belgium, 2003)	Own isolate
ST189-CF	LMG 29312, BCC0208	189	CF patient (Canada, 1999)	E. Mahenthiralingam
ST287-ENV	LMG 29306, J2947	287	Rhizosphere soil (United Kingdom, 2000)	J.R.W. Govan
ST287-CF	LMG 29311, BCC0059	287	CF patient (Canada, 1995)	E. Mahenthiralingam
ST650-ENV	LMG 29308	650	Pond water (Belgium, 2003)	Own isolate
ST650-CF	LMG 29310, Q113	650	CF patient (Germany, 2010)	B. Kahl

LMG, BCCM/LMG Bacteria Collection, Laboratory of Microbiology, Ghent University, Ghent, Belgium. CF, cystic fibrosis; ENV, environmental; ST, multilocus sequence type.

### Genome sequencing, assembly and annotation

High-quality DNA was prepared using Qiagen Genomic tips (20/G) and genomes were sequenced using the P5-C3 chemistry on the PacBio SMRT II platform of the Department of Genetics and Genomic Sciences of the Icahn School of Medicine at Mount Sinai (New York, USA). One SMRT cell per isolate was sequenced, except for isolates ST189-CF and ST287-ENV for which a second SMRT cell was run to increase the quality of the raw data. PacBio reads were assembled in the sequencing center using the SMRT analysis software (including HGAP3 and Quiver) and contigs were ordered against the complete reference genome of *B. multivorans* strain ATCC 17616 (PRJNA17407) using Mauve [19]. We further polished the assemblies in five steps. The first step consisted of removing spurious contigs that were small in size, had a low coverage and resulted in a highest BLAST hit with the primary chromosome of its own genome [20]. Reads were mapped using pbalign and QC reports were created based on the resulting BAM files using Qualimap [21]. Contigs smaller than 20 kb and with less than 20x coverage or a high variation (SD) in coverage were discarded. In step two, read mappings were used to further polish the contigs using Pilon [22] with default parameters. In step three, contigs with overlapping ends were merged using Gap5 [23] to exclude artificially duplicated regions, often including many frameshifts and fragmented open reading frames. In step four, the duplicated ends of circular contigs were trimmed using Gepard [24] and Gap5 as these duplications were a consequence of the circular nature of the replicons in combination with the long-read sequencing technology [25]. Importantly, this artificial duplication of contig ends not only resulted in a highly variable rRNA copy number, but also falsely excluded genes from the ortholog dataset because they were artificially duplicated. Since the merging of overlapping ends by Gap5 might be imperfect, we ran Quiver in a final polishing step. The PacBio sequencing reads of one SMRT cell resulted in a coverage ranging from 76x to 119x.

Annotation was performed using Prokka v1.11 [26] with a genus-specific database based on reference genomes from the *Burkholderia* Genome database (http://beta.burkholderia.com/, accessed March 2015) [27]. The annotated genome assemblies were submitted to the European Nucleotide Archive and are publicly available through the GenBank/EMBL/DDBJ accession numbers FKJT01000000, FKJS01000000, FKJU01000000, FKJP01000000, FKJV01000000, FKJW01000000, FKJX01000000 and FKJY01000000. The original PacBio sequencing data were submitted to the Sequence Read Archive and are publicly available through the accession numbers ERX1955257, ERX1955260, ERX1955324, ERX1955331, ERX1955371, ERX1955980, ERX1955987 and ERX1955995.

The genome sequence of *B. multivorans* strain ATCC 17616 (PRJNA17407) was included as a reference in all further analyses. A multiple genome alignment was performed using Mauve [28] to assess the basic genome structure.

### Analysis of protein-coding genes and ortholog identification

We mapped for each protein-coding gene (CDS) on which chromosome it was located and to which cluster of orthologous groups (COG) [29] it belonged (S5 Table). COGs were assigned by a reversed position-specific BLAST (RPSBLAST v2.2.29+) with an e-value cut-off of 1E-3 against the NCBI conserved domain database (CDD v3.14). Orthologous genes were identified in the eight *B. multivorans* genomes of the present study and the ATCC 17616 reference strain using custom perl scripts (https://github.com/hatcherunh/GeneFamilyAnalysis) as described previously [30, 31]. In short, homologs were identified as reciprocal best BLAST hits with a normalized bit score (bit score of hit/bit score of self-hit, see [31]), providing an empirically determined taxon-specific threshold. A CDS was defined to be non-orthologous if no orthologs were found in the dataset. The putative panorthologs (i.e. single-copy orthologous genes conserved in all genomes) were computed while varying the bit score threshold from 0.1 to 0.9 in 0.1 increments and the largest set of panorthologs was selected. For each orthologous protein family, the consensus chromosome location and COG were determined (S6 Table). Conflicts in COG mapping were resolved by the majority rule.

### Phylogenomic analysis

The whole-genome phylogeny (of the eight *B. multivorans* genomes of the present study and the ATCC 17616 reference strain) was calculated based on the sequences of the panorthologs as described previously [15]. In short, amino acid sequences were aligned using MUSCLE [32] and translated back to the respective nucleotide sequences using T-Coffee [33]. Nucleotide alignments were trimmed using trimAl [34] by removing positions with gaps in more than 50% of the sequences, and were subsequently concatenated to construct a maximum likelihood tree using RaXML v7.4.2 [35] with the GTRGAMMA substitution model and 1000 rapid bootstrap analyses.

In a second approach, the presence/absence matrix of all orthologs was used in a discrete character-state parsimony analysis using pars from the PHYLIP package [36] to assess the relatedness of the genomes in terms of gene content.

### Comparison of *B. multivorans* and *B. cenocepacia* COG profiles

Complete genome sequences of *Burkholderia cenocepacia* strains J2315, H111, K56-2Valvano, AU1054, HI2424 and MC0-3 were downloaded from the *Burkholderia* Genome database (http://beta.burkholderia.com/) [27]. COG mapping of *B. cenocepacia* CDS was performed as described above for *B. multivorans*. The number of CDS per COG category for each species (*B. multivorans* versus *B. cenocepacia*) was counted and the distributions were compared using Pearson chi-square analysis.

### Data visualization and statistical analyses

Data visualization and statistical analyses were performed using RStudio with R v3.2.3. Pearson’s chi-square analyses were used to test the association between different sets of categorical variables. When a significant relationship was found between two variables, we further examined the standardized Pearson residuals. Standardized Pearson residuals with high absolute values indicate a lack of fit of the null hypothesis of independence in each cell [37] and thus indicate observed cell frequencies in the contingency table that are significantly higher or lower than expected based on coincidence. In case multiple COG categories were registered for the same COG, each COG category was counted separately for Pearson chi-square analysis on COG categories. For the 198 CDS that were involved in the translocation within the ST650-CF isolate from the primary to the secondary chromosome, the consensus chromosome mapping was set to the primary chromosome for Pearson chi-square analysis on chromosome distribution.

## Results

### The genomic structure of *B. multivorans* is highly conserved

The final assemblies produced closed genomes for five of the eight sequenced *B. multivorans* isolates. The genomes were 6.2-6.9 Mb in size with a G+C content of ˜67 mol% and the number of predicted CDS ranged from 5,415 to 6,155 CDS per genome (Table 2). No clustered regularly interspaced short palindromic repeats (CRISPRs) were identified. Each of the genomes contained one tmRNA and 75-79 tRNAs.

**Table 2:** *B. multivorans* genome characteristics.

Isolate	Contigs	Size (bp)	%GC	C1	C2	C3	Plasmid	Total CDS	Orthologous CDS	Non-orthologous CDS
ST180-ENV	4	6,464,081	67.1	1	2	3	4	5,794	5,271	523
ST180-CF	4	6,296,736	67.3	1	2	3	4	5,551	5,266	285
ST189-ENV	3	6,223,431	67.3	1	2	3	-	5,467	5,144	323
ST189-CF	13	6,157,395	67.3	1-5	6-9	10-13	-	5,415	5,132	283
ST287-ENV	4	6,559,547	67.2	1-2	3	4	-	5,800	5,494	306
ST287-CF	6	6,857,684	67.0	1-4	5	6	-	6,155	5,505	650
ST650-ENV	3	6,322,929	67.2	1	2	3	-	5,594	5,275	319
ST650-CF	3	6,308,820	67.2	1	2	3	-	5,599	5,250	349
ATCC 17616	4	7,008,622	66.7	1	2	3	4	6,258	4,893	1,365

C1, C2 and C3: contigs mapping to chromosome 1, 2 and 3 of *B. multivorans* strain ATCC 17616, respectively; bp, base pairs.

The multiple genome alignment of the examined *B. multivorans* STs (S3 Fig) revealed a highly conserved genomic structure with three chromosomes (from here on referred to as C1, C2 and C3). C1, C2 and C3 were on average 3.4 Mb, 2.4 Mb and 0.6 Mb in size. Both ST180 isolates harbored one contig that did not map onto the reference genome of ATCC 17616. These contigs were 22,339 and 28,809 bp in size, did not contain any rRNA genes, had a G+C content of 58 mol% and were therefore considered plasmids. Both plasmids contained genes for an initiator repB protein, an AsnC transcriptional regulator, a cobyrinic acid a,c-diamide synthase (*parA* homologue), multiple integrases and several hypothetical proteins. The multiple genome alignment also revealed a fairly large translocation (207 genes, 198 CDS) within the ST650-CF isolate from C1 to C2 that was delimited by rRNA operons at both ends. All isolates except ST650-CF contained 3, 1 and 1 rRNA copies on C1, C2 and C3, respectively. As a result of the translocation, isolate ST650-CF contained 2, 2 and 1 rRNA copies on C1, C2 and C3, respectively.

### ST predicts genomic lineage

Orthologous genes were identified to determine the conserved genome content of *B. multivorans*. The ortholog analysis identified 6,254 homologous protein families (S6 Table) comprising 47,230 CDS in total (Table 2). The largest set of panorthologs, i.e. orthologs conserved in all nine *B. multivorans* genomes and present as single copies, was found at a reciprocal best bit score threshold of 0.7 (see Methods section) and comprised 4,503 ortholog families.

The frequency of orthologous versus non-orthologous CDS (i.e. CDS with versus without orthologs in the dataset) varied significantly per isolate (X^2^(8) = 1829.6, p<0.001) and ST (X^2^(3) = 67.3, p<0.001), but not isolation source (p>0.05). The genomes of isolates ST287-CF and ATCC 17616 were significantly enriched with non-orthologous CDS, while those of ST180-CF, ST287-ENV, ST650-ENV and both ST189 isolates were significantly deprived in non-orthologous CDS (Table 2 and S1 Table). Analysis of the relationship between orthologous versus non-orthologous CDS and ST showed that the ST287 genomes were significantly enriched with non-orthologous CDS, while the ST189 and ST650 genomes were significantly deprived in non-orthologous CDS (S2 Table).

**Fig 1:**
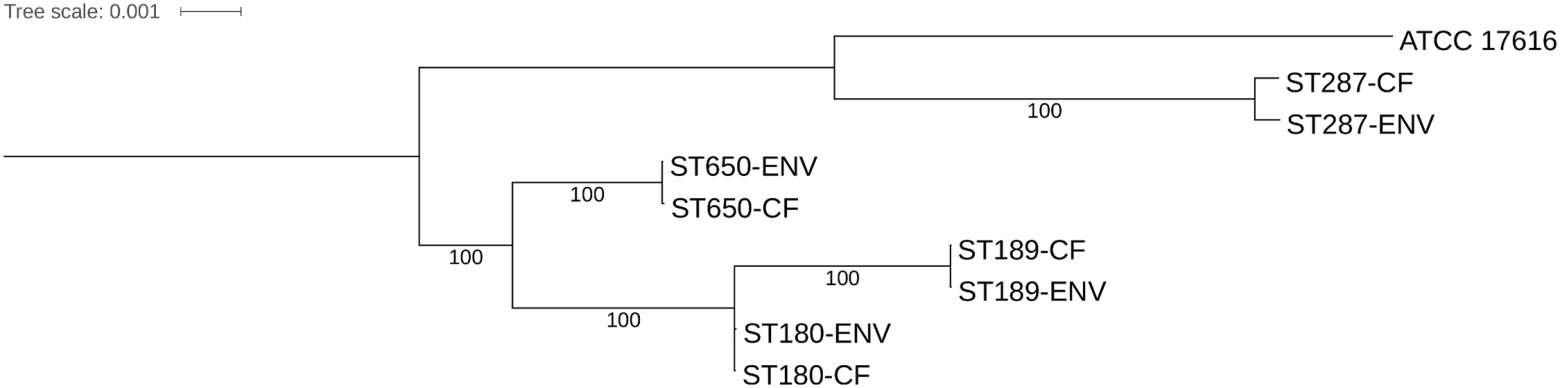
Phylogenomic analysis showing the relatedness of the genomes in terms of sequence divergence of the panorthologs. The maximum likelihood tree was inferred using the GTRGAMMA substitution model and is based on a concatenated nucleotide alignment of 4,503 CDS (4,457,847 positions). The percentage of replicate trees in which the associated taxa clustered together in the bootstrap analyses (1,000 replicates) are shown next to the branches. Scale bar represents number of substitutions per site. The tree was rooted on the branch with the largest branch length.

The ortholog dataset enabled two subsequent analyses of strain phylogeny. In the first approach, a whole-genome phylogeny was obtained based on nucleotide sequence divergence of the panorthologs (Fig 1). In the second approach, the presence/absence matrix of the ortholog families was used to assess the relatedness of the genomes in terms of gene content using parsimony (S1 Fig). These analyses both demonstrated that the ST, and not the isolation source, of the *B. multivorans* isolates predicted their phylogeny and gene content. This finding demonstrated that isolates with the same ST represent the same genomic lineage, irrespective of their isolation source.

### Orthologous genes are enriched on C2 and are involved in carbohydrate metabolism and transport

**Fig 2:**
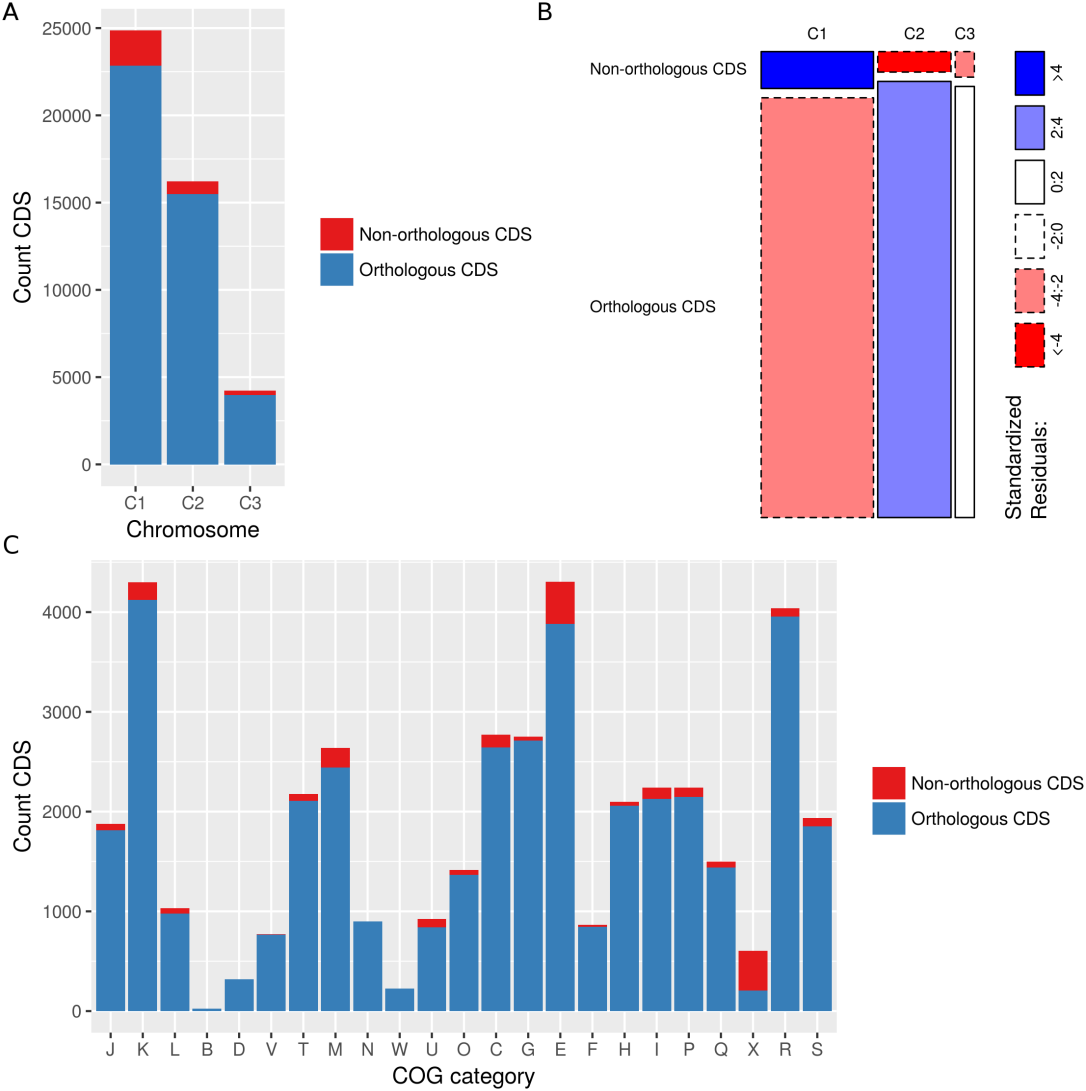
The frequency of orthologous versus non-orthologous CDS varies among chromosomes and COG categories. Bar plots show the number of orthologous and non-orthologous CDS per chromosome (X^2^(2) = 213.4, p<0.001) **(a)** and COG category (X^2^(22) = 5101.2, p<0.001) **(c)**. Mosaic plots show the standardized residuals of the Pearson chi-square analysis for the number of orthologous and non-orthologous CDS per chromosome **(b)**. Solid and dashed boundaries represent positive and negative residuals, respectively. Rectangles are colored only if the standardized residual is significant at p<0.05 (outside ±1.96). COG categories: J, translation, ribosomal structure and biogenesis; K, transcription; L, replication, recombination and repair; B, chromatin structure and dynamics; D, cell cycle control, cell division, chromosome partitioning; V, defense mechanisms; T, signal transduction mechanisms; M, cell wall/membrane/envelope biogenesis; N, cell motility; W, extracellular structures; U, intracellular trafficking, secretion, and vesicular transport; O, posttranslational modification, protein turnover, chaperones; X, mobilome: prophages, transposons; C, energy production and conversion; G, carbohydrate transport and metabolism; E, amino acid transport and metabolism; F, nucleotide transport and metabolism; H, coenzyme transport and metabolism; I, lipid transport and metabolism; P, inorganic ion transport and metabolism; Q, secondary metabolites biosynthesis, transport and catabolism; R, general function prediction only; S, function unknown.

Because the fraction of genes that are involved in housekeeping functions varies among the chromosomes [17], we mapped the chromosome location of each CDS (S5 Table). Consistent with the average chromosome size, the total number of CDS was highest on C1 (27,813 CDS), followed by C2 (18,565 CDS) and C3 (5,047 CDS). The plasmid of the ST180 isolates harbored 208 CDS, of which 206 were non-orthologous CDS. The translocation within the ST650-CF isolate from C1 to C2 comprised 198 CDS, of which 13 were non-orthologous CDS. The frequency of orthologous versus non-orthologous CDS varied significantly among the different chromosomes (X^2^(2) = 213.4, p<0.001) (Fig 2A). C1 was significantly enriched with non-orthologous CDS, while C2 was significantly enriched with orthologous CDS (Fig 2B).

To identify biological functions that were over- or underrepresented, we assigned each CDS to a COG (S5 Table). Roughly 80% of the CDS (41,520 CDS in total) could be assigned to a COG and its associated COG functional category. The frequency of orthologous versus non-orthologous CDS varied significantly among the different COG categories (X^2^(22) = 5101.2, p<0.001) (Fig 2C). The non-orthologous CDS were significantly enriched in the COG categories cell wall/membrane/envelope biogenesis (M), intracellular trafficking, secretion and vesicular transport (U), amino acid transport and metabolism (E) and mobilome (X), while the orthologous CDS were significantly enriched in the COG categories carbohydrate transport and metabolism (G) and general function prediction only (R) (Table 3).

**Table 3:** The frequency of orthologous versus non-orthologous CDS varies among the COG categories.

		Orthologous CDS		Non-orthologous CDS	
**Information storage and processing**					
J	Translation, ribosomal structure and biogenesis		1814 (0.789)	−	65 (-3.359)
K	Transcription		4122 (0.766)	−	176 (-3.258)
L	Replication, recombination and repair		977 (-0.032)		55 (0.137)
B	Chromatin structure and dynamics		24 (0.263)		0 (-1.121)
**Cellular processes and signaling**					
D	Cell cycle control, cell division, chromosome partitioning		319 (0.848)	−	2 (-3.610)
V	Defense mechanisms		765 (1.199)	−	8 (-5.101)
T	Signal transduction mechanisms		2108 (0.988)	−	69 (-4.207)
M	Cell wall/membrane/envelope biogenesis		2441 (-1.161)	+	196 (4.941)
N	Cell motility		898 (1.510)	−	3 (-6.429)
W	Extracellular structures		225 (0.806)	−	0 (-3.431)
U	Intracellular trafficking, secretion, and vesicular transport		838 (-1.241)	+	85 (5.283)
O	Posttranslational modification, protein turnover, chaperones		1367 (0.711)	−	48 (-3.025)
**Metabolism**					
C	Energy production and conversion		2641 (0.329)		128 (-1.401)
G	Carbohydrate transport and metabolism	+	2710 (2.016)	−	41 (-8.579)
E	Amino acid transport and metabolism	−	3880 (-3.142)	+	426 (13.373)
F	Nucleotide transport and metabolism		845 (0.916)	−	19 (-3.897)
H	Coenzyme transport and metabolism		2058 (1.521)	−	42 (-6.475)
I	Lipid transport and metabolism		2127 (0.091)		113 (-0.387)
P	Inorganic ion transport and metabolism		2148 (0.567)	−	91 (-2.415)
Q	Secondary metabolites biosynthesis, transport and catabolism		1442 (0.543)	−	58 (-2.311)
**Poorly characterized**					
R	General function prediction only	+	3954 (2.026)	−	86 (-8.623)
S	Function unknown		1851 (0.358)		86 (-1.524)
**Mobile elements**					
X	Mobilome: prophages, transposons	−	207 (-15.300)	+	398 (65.117)

Pearson chi-square analysis testing the independence of gene conservation (orthologous vs. non-orthologous CDS) and COG category (X^2^(22) = 5101.2, p<0.001). Each cell in the contingency represents the observed frequency and standardized residual (in between brackets) and is preceded by + or − if the standardized residual is >1.96 or <-1.96, respectively, and significant at p<0.05.

### Each ST harbors unique orthologs

**Fig 3:**
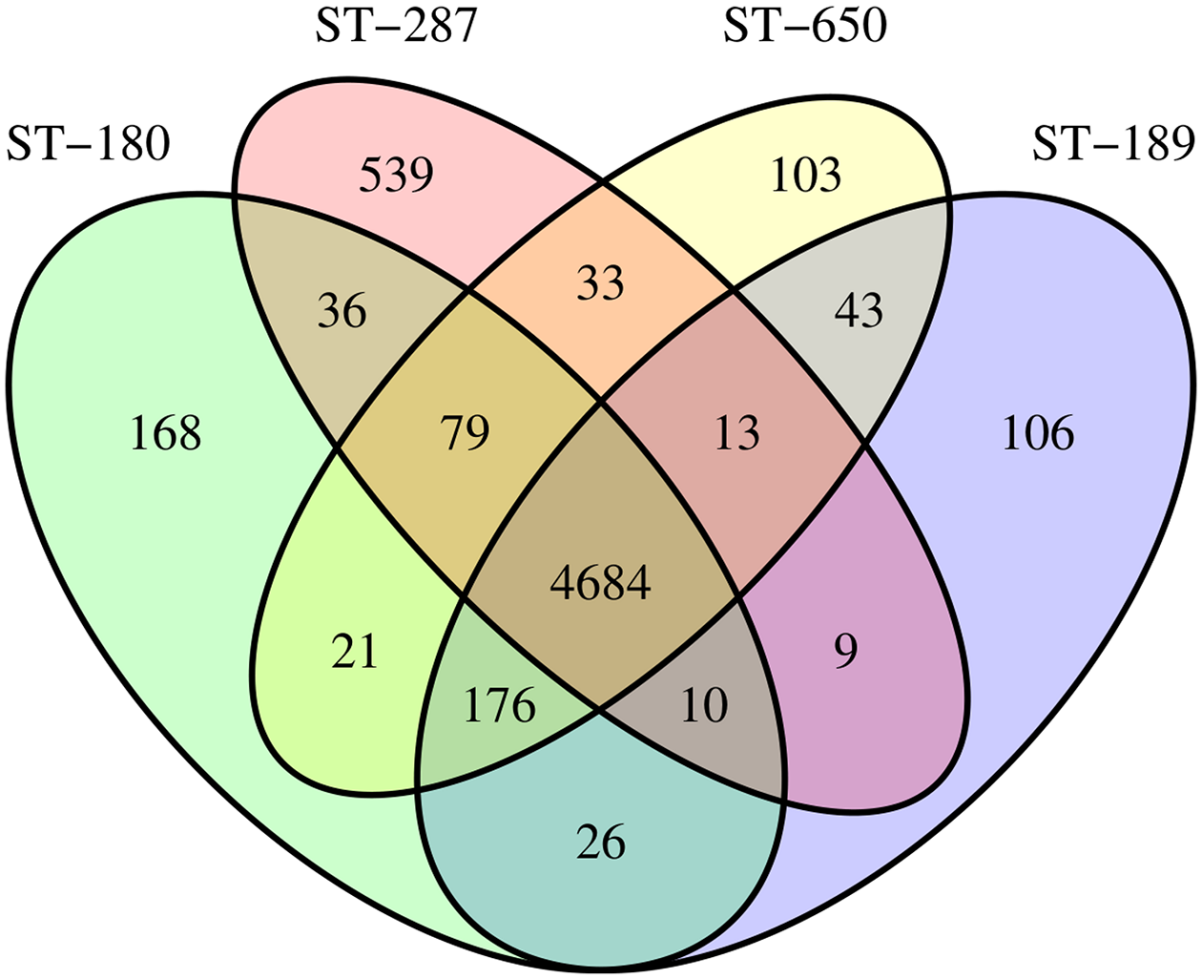
Venn diagram showing the number of core and ST-specific ortholog families. ST, multilocus sequence type.

For each ortholog family we examined whether it was present in all eight isolates (i.e. showed core specificity), specific for isolates of one or more STs, specific for isolates of a specific source or randomly present (S6 Table). None of the ortholog families was present in all four isolates of a specific source (CF vs. ENV), but a small number of ortholog families were present in only one, two or three isolates from the same source, thus leaving five relevant specificity groups: core (n = 4,684), ST (n = 1,362), CF-only (n = 38), ENV-only (n = 51) and random (n = 119). The Venn diagram (Fig 3) visualizes the number of ortholog families in the core and ST specificity groups (n = 6,046) and shows that each ST harbors 103-539 orthologs that were not present in any other ST.

### ST-specific orthologs are enriched on C2 and C3 and are involved in defense mechanisms and secondary metabolism

Based on the chromosome and COG mapping for the individual CDS, we mapped the consensus chromosome location and COG category for each ortholog family (S6 Table). Consistent with the average chromosome size, the number of orthologs was highest on C1 (3,242), followed by C2 (2,264) and C3 (710). For 37 ortholog families there was a conflict in chromosome mapping, and 1 ortholog was located on the plasmid of the ST180 isolates. COGs and their associated COG functional category could be assigned to 4,896 of the 6,254 ortholog families.

**Fig 4:**
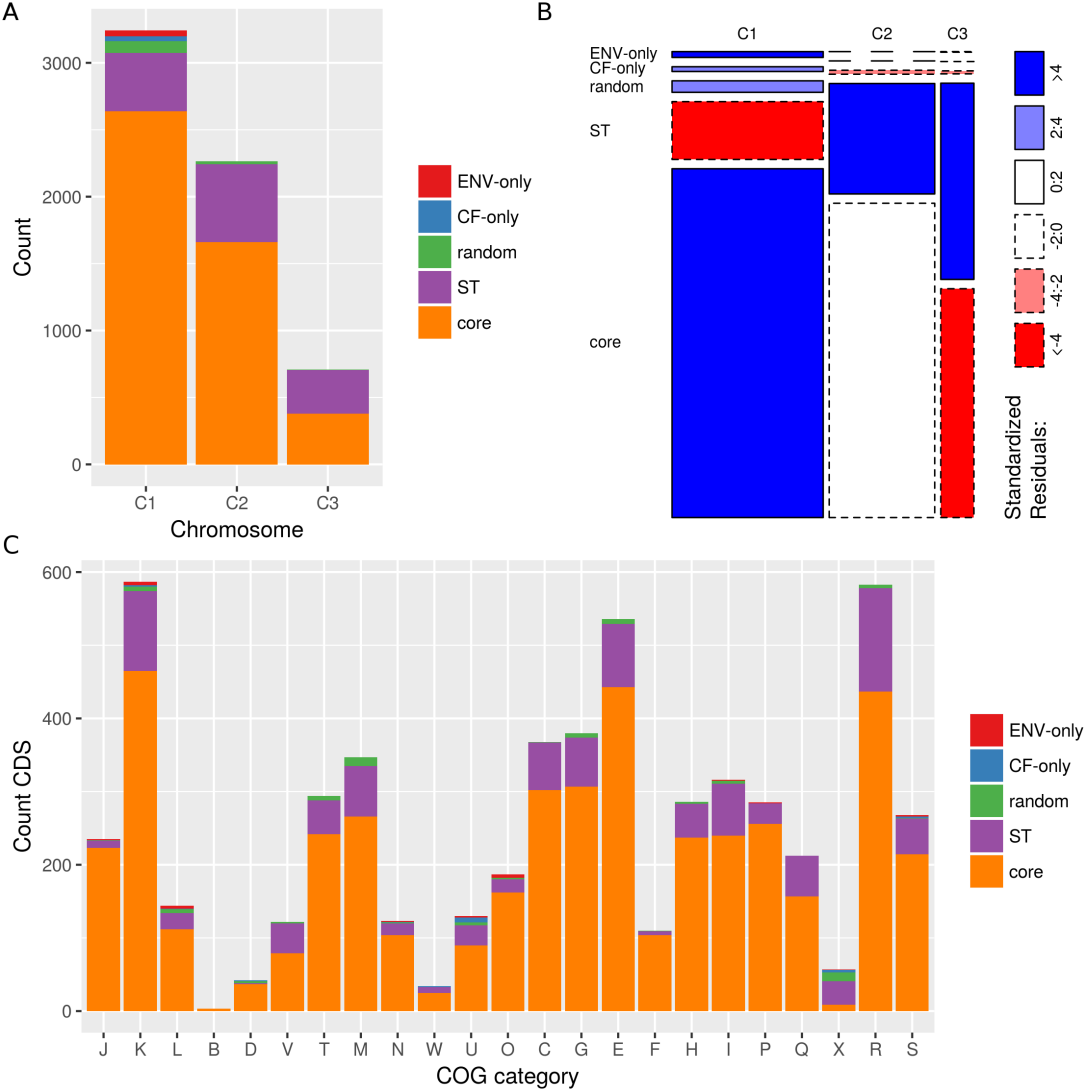
Ortholog specificity varies among chromosomes and COG categories. Bar plots show the number of orthologs per specificity group for different chromosomes (X^2^(8) = 469.8, p<0.001) **(a)** and COG categories (X^2^(88) = 649.8, p<0.001) **(c)**. Mosaic plots show the standardized residuals of the Pearson chi-square analysis on the number of orthologs per specificity group per chromosome **(b)**. Solid and dashed boundaries represent positive and negative residuals, respectively. Rectangles are colored only if the standardized residual is significant at p<0.05 (outside ±1.96). COG categories: J, translation, ribosomal structure and biogenesis; K, transcription; L, replication, recombination and repair; B, chromatin structure and dynamics; D, cell cycle control, cell division, chromosome partitioning; V, defense mechanisms; T, signal transduction mechanisms; M, cell wall/membrane/envelope biogenesis; N, cell motility; W, extracellular structures; U, intracellular trafficking, secretion, and vesicular transport; O, posttranslational modification, protein turnover, chaperones; X, mobilome: prophages, transposons; C, energy production and conversion; G, carbohydrate transport and metabolism; E, amino acid transport and metabolism; F, nucleotide transport and metabolism; H, coenzyme transport and metabolism; I, lipid transport and metabolism; P, inorganic ion transport and metabolism; Q, secondary metabolites biosynthesis, transport and catabolism; R, general function prediction only; S, function unknown.

The specificity of the ortholog families varied significantly among the chromosomes (X^2^(8) = 469.8, p<0.001) (Fig 4A) and COG categories (X^2^(88) = 649.8, p<0.001) (Fig 4C). C2 and C3 were significantly enriched with ST-specific orthologs, while C1 was significantly enriched with orthologs belonging to the specificity groups core, random, CF-only and ENV-only (Fig 4B). The ST-specific orthologs were significantly enriched in the COG categories defense mechanisms (V), secondary metabolites biosynthesis, transport and catabolism (Q), mobilome (X) and general function prediction only (R) (Table 4).

**Table 4:** Ortholog specificity varies among the COG categories.

		Core		ST		CF-only		ENV-only		Random	
**Information storage and processing**											
J	Translation, ribosomal structure and biogenesis	+	223 (2.570)	−	10 (-4.947)		0 (-0.865)		1 (0.044)		1 (-1.306)
K	Transcription		465 (-0.187)		109 (0.375)		2 (0.095)		5 (1.688)		6 (-0.864)
L	Replication, recombination and repair		112 (-0.286)		22 (-0.748)		1 (0.799)	+	4 (4.458)	+	5 (2.013)
B	Chromatin structure and dynamics		3 (0.389)		0 (-0.733)		0 (-0.098)		0 (-0.111)		0 (-0.209)
**Cellular processes and signaling**											
D	Cell cycle control, cell division, chromosome partitioning		37 (0.594)	−	1 (-2.378)	+	1 (2.368)		0 (-0.414)	+	3 (3.061)
V	Defense mechanisms		79 (-1.872)	+	41 (4.095)		0 (-0.623)		0 (-0.705)		2 (0.172)
T	Signal transduction mechanisms		242 (0.461)		46 (-0.919)		0 (-0.968)		0 (-1.094)		6 (0.839)
M	Cell wall/membrane/envelope biogenesis		266 (-0.677)		69 (0.867)		0 (-1.052)		0 (-1.189)	+	12 (3.102)
N	Cell motility		104 (0.576)		16 (-1.286)		1 (0.971)		1 (0.705)		1 (-0.588)
W	Extracellular structures		25 (-0.416)		8 (0.774)	+	1 (2.709)		0 (-0.372)		0 (-0.703)
U	Intracellular trafficking, secretion, and vesicular transport		90 (-1.362)		27 (0.769)	+	7 (10.233)	+	2 (2.022)		4 (1.538)
O	Posttranslational modification, protein turnover, chaperones		162 (1.028)	−	18 (-2.678)		0 (-0.772)	+	5 (4.858)		2 (-0.434)
**Metabolism**											
C	Energy production and conversion		302 (0.463)		65 (-0.114)		0 (-1.083)		0 (-1.224)		1 (-1.879)
G	Carbohydrate transport and metabolism		307 (0.192)		67 (-0.130)		0 (-1.100)		0 (-1.244)		6 (0.206)
E	Amino acid transport and metabolism		443 (0.710)		86 (-1.023)		0 (-1.307)		0 (-1.477)		7 (-0.280)
F	Nucleotide transport and metabolism		104 (1.717)	−	5 (-3.313)		0 (-0.592)		0 (-0.669)		1 (-0.472)
H	Coenzyme transport and metabolism		237 (0.560)		46 (-0.731)		0 (-0.955)		0 (-1.079)		3 (-0.565)
I	Lipid transport and metabolism		240 (-0.787)		71 (1.913)		0 (-1.003)		1 (-0.253)		4 (-0.274)
P	Inorganic ion transport and metabolism		256 (1.873)	−	28 (-3.227)		0 (-0.953)		1 (-0.149)	−	0 (-2.034)
Q	Secondary metabolites biosynthesis, transport and catabolism		157 (-0.953)	+	55 (2.762)		0 (-0.822)		0 (-0.929)		0 (-1.754)
**Poorly characterized**											
R	General function prediction only		437 (-1.337)	+	141 (3.577)		0 (-1.363)		0 (-1.541)		5 (-1.190)
S	Function unknown		214 (-0.010)		49 (0.143)		2 (1.240)		2 (0.870)		1 (-1.465)
**Mobile elements**											
X	Mobilome: prophages, transposons	−	9 (-5.415)	+	32 (6.818)	+	3 (6.613)		1 (1.594)	+	12 (12.283)

Pearson chi-square analysis testing the independence of ortholog specificity and COG category (X^2^(88) = 469.8, p<0.001). Each cell in the contingency represents the observed frequency and standardized residual (in between brackets) and is preceded by + or − if the standardized residual is >1.96 or <-1.96, respectively, and significant at p<0.05.

### ST287 harbors extra orthologous and non-orthologous genes

Both ST287 genomes were considerably larger and contained a higher number of CDS (Table 2), suggesting that this genomic lineage contains extra genes. ST287 was not only enriched with non-orthologous CDS (S2 Table) but also harbored 539 orthologs that were not present in the other three STs (Fig 3), showing that the extra genes in ST287 are both orthologous and non-orthologous CDS. A similar trend was observed for the ST287-specific orthologs as compared to the ST-specific orthologs in general (Table 4), as they were enriched in the same COG categories.

### C1 is enriched with orthologs showing CF and ENV specificity

C1 was enriched with orthologs that were present in only one, two or three isolates from the same source (CF-only or ENV-only) (Fig 4A and 4B). CF-only orthologs were significantly enriched in the COG categories cell cycle control, cell division and chromosome partitioning (D), extracellular structures (W), intracellular trafficking, secretion and vesicular transport (U) and mobilome (X), while ENV-only orthologs were significantly enriched in replication, recombination and repair (L), intracellular trafficking, secretion and vesicular transport (U) and posttranslational modification, protein turnover and chaperones (O) (Fig 4C and Table 4).

Additionally, C1 was also enriched with orthologs showing random specificity (Fig 4A and 4B) and these orthologs with random specificity were significantly enriched in the COG categories replication, recombination and repair (L), cell cycle control, cell division and chromosome partitioning (D), cell wall/membrane/envelope biogenesis (M) and mobilome (X) (Fig 4C and Table 4).

### Comparison of *B. multivorans* and *B. cenocepacia* average COG profiles

During the past two decades, *B. multivorans* and *B. cenocepacia* have been the most prevalent Bcc pathogens in CF. Historically, *B. cenocepacia* strains have been responsible for large epidemics within the CF community and are often extremely virulent [38]. In contrast, only a limited number of *B. multivorans* outbreak strains were described and *B. multivorans* is generally considered a less virulent Bcc pathogen as compared to *B. cenocepacia* [17]. To examine the species-specific genome content of *B. multivorans*, we compared its average COG profile to that of *B. cenocepacia*. Generally, *B. cenocepacia* genomes contained more genes (6,477-7,116 CDS per genome) (S3 Table) than *B. multivorans* genomes (5,415-6,155 CDS per genome) (Table 2) and more CDS per COG category (S2 Fig). We compared the average COG profile of the two species by calculating the average number of CDS per genome in each COG category and by comparing these distributions. The distribution of CDS among COG categories varied significantly between the two species (X^2^(22) = 102.9, p<0.001) (S2 Fig). *B. cenocepacia* genomes harbor significantly more CDS in the COG categories transcription (K), defense mechanisms (V) and general function prediction only (R) and significantly less in translation, ribosomal structure and biogenesis (J) and replication, recombination and repair (L). Conversely, *B. multivorans* genomes harbor significantly more CDS in the COG categories replication, recombination and repair (L) and less in transcription (K) (Table 5).

**Table 5:** The distribution of *B. multivorans* versus *B. cenocepacia* CDS varies among COG categories.

		*B. cenocepacia*		*B. multivorans*	
**Information storage and processing**					
J	Translation, ribosomal structure and biogenesis	−	1528 (-2.166)		2118 (1.931)
K	Transcription	+	4314 (3.883)	−	4866 (-3.462)
L	Replication, recombination and repair	−	809 (-2.797)	+	1206 (2.494)
B	Chromatin structure and dynamics		21 (-0.057)		27 (0.051)
**Cellular processes and signaling**					
D	Cell cycle control, cell division, chromosome partitioning		261 (-1.082)		369 (0.964)
V	Defense mechanisms	+	800 (2.046)		880 (-1.825)
T	Signal transduction mechanisms		2032 (0.726)		2482 (-0.647)
M	Cell wall/membrane/envelope biogenesis		2438 (0.658)		2993 (-0.587)
N	Cell motility		715 (-1.808)		1012 (1.612)
W	Extracellular structures		226 (0.949)		253 (-0.846)
U	Intracellular trafficking, secretion, and vesicular transport		750 (-1.798)		1058 (1.603)
O	Posttranslational modification, protein turnover, chaperones		1251 (-0.421)		1607 (0.376)
**Metabolism**					
C	Energy production and conversion		2357 (-1.677)		3151 (1.496)
G	Carbohydrate transport and metabolism		2519 (0.619)		3098 (-0.552)
E	Amino acid transport and metabolism		3694 (-1.403)		4840 (1.251)
F	Nucleotide transport and metabolism		747 (-0.603)		977 (0.538)
H	Coenzyme transport and metabolism		1854 (-0.344)		2364 (0.297)
I	Lipid transport and metabolism		2090 (1.130)		2513 (-1.008)
P	Inorganic ion transport and metabolism		1883 (-1.820)		2550 (1.623)
Q	Secondary metabolites biosynthesis, transport and catabolism		1367 (0.495)		1678 (-0.441)
**Poorly characterized**					
R	General function prediction only	+	3843 (1.965)		4562 (-1.752)
S	Function unknown		1685 (-0.887)		2202 (0.791)
**Mobile elements**					
X	Mobilome: prophages, transposons		630 (0.830)		746 (-0.740)

Pearson chi-square analysis testing the independence of species and COG category (X^2^(22) = 102.9, p<0.001). Each cell in the contingency represents the observed frequency and standardized residual (in between brackets) and is preceded by + or − if the standardized residual is >1.96 or <-1.96, respectively, and significant at p<0.05.

Finally, we searched for COGs that were exclusively present in either *B. multivorans* or *B. cenocepacia* genomes. In total, 124 COGs were exclusively present in one or more *B. multivorans* genomes, but only 21 COGs were uniquely present in all nine of the *B. multivorans* genomes (Table 6). Conversely, 204 COGs were exclusively present in one or more *B. cenocepacia* genomes, but only 72 COGs were uniquely present in all six of the *B. cenocepacia* genomes (S4 Table).

**Table 6:** *B. multivorans*-specific COGs.

COG	COG name	COG category
COG0062	NAD(P)H-hydrate repair enzyme Nnr, NAD(P)H-hydrate epimerase domain	F
COG0645	Predicted kinase	R
COG1585	Membrane protein implicated in regulation of membrane protease activity	O
COG2312	Erythromycin esterase homolog	Q
COG4121	tRNA U34 5-methylaminomethyl-2-thiouridine-forming methyltransferase MnmC	J
COG5567	Predicted small periplasmic lipoprotein YifL (function unknown)	S
COG5615	Uncharacterized membrane protein	S
COG2519	tRNA A58 N-methylase Trm61	J
COG2905	Signal-transduction protein containing cAMP-binding, CBS, and nucleotidyltransferase domains	T
COG3059	Uncharacterized membrane protein YkgB	S
COG3095	Chromosome condensin MukBEF, MukE localization factor	D
COG3220	Uncharacterized conserved protein, UPF0276 family	S
COG4823	Abortive infection bacteriophage resistance protein	V
COG5453	Uncharacterized protein	S
COG1107	Archaea-specific RecJ-like exonuclease, contains DnaJ-type Zn finger domain	L
COG1140	Nitrate reductase beta subunit	CP
COG2180	Nitrate reductase assembly protein NarJ, required for insertion of molybdenum cofactor	CPO
COG2181	Nitrate reductase gamma subunit	CP
COG2202	PAS domain	T
COG2427	Uncharacterized conserved protein YjgD, DUF1641 family	S
COG5013	Nitrate reductase alpha subunit	CP

COG categories: J, translation, ribosomal structure and biogenesis; K, transcription; L, replication, recombination and repair; B, chromatin structure and dynamics; D, cell cycle control, cell division, chromosome partitioning; V, defense mechanisms; T, signal transduction mechanisms; M, cell wall/membrane/envelope biogenesis; N, cell motility; W, extracellular structures; U, intracellular trafficking, secretion, and vesicular transport; O, posttranslational modification, protein turnover, chaperones; X, mobilome: prophages, transposons; C, energy production and conversion; G, carbohydrate transport and metabolism; E, amino acid transport and metabolism; F, nucleotide transport and metabolism; H, coenzyme transport and metabolism; I, lipid transport and metabolism; P, inorganic ion transport and metabolism; Q, secondary metabolites biosynthesis, transport and catabolism; R, general function prediction only; S, function unknown.

## Discussion

Of the 20 formally named species within the Bcc, *B. multivorans* and *B. cenocepacia* are generally the most prevalent Bcc species in CF [1]. Historically, *B. cenocepacia* strains have been responsible for large epidemics within the CF community and are often extremely virulent [38]. While infection control measures reduced patient-to-patient transmission and thereby the prevalence of *B. cenocepacia*, *B. multivorans* is characterized by a limited person-to-person transmission and subsequently emerged as the most prevalent Bcc pathogen in many countries [1–7]. The low number of outbreaks caused by *B. multivorans* [39–41] and the fact that isolates from CF patients commonly represent unique strains suggest that strains are acquired from non-human sources such as the natural environment [8]. To examine the extent to which the ST of *B. multivorans* isolates from CF versus environmental samples explains their genomic content and functionality, we selected four pairs of *B. multivorans* isolates for whole-genome sequencing, representing distinct STs and consisting of one CF and one environmental isolate each.

MLST is a well-established method for studying the population structure of Bcc organisms [11, 12] and Baldwin *et al.* [8] previously reported the occurrence of *B. multivorans* STs that were globally distributed. Recently, whole-genome sequencing of *B. pseudomallei* revealed that the unexpected occurrence of two *B. pseudomallei* STs on two continents was due to homoplasy [42]. In contrast, the present study demonstrated that the ST predicted both phylogeny and gene content of *B. multivorans* isolates (Fig 1 and S1 Fig) and hence corroborates the use of MLST for epidemiological surveillance of Bcc bacteria.

The clinical isolates of ST189 and ST287 were obtained from samples of Canadian CF patients, but the environmental isolates of these STs were soil isolates from Belgium and the United Kingdom, respectively (Table 1). Yet, our analyses showed that, despite this transatlantic barrier, each *B. multivorans* genomic lineage was defined by its ST, harboring a highly conserved set of genes (S1 Fig). Moreover, isolates belonging to the same ST were isolated up to eleven years apart (Table 1). Finally, searching the Bcc PubMLST database (http://pubmlst.org/bcc/) [13] for additional isolates of the studied STs (Table 1) revealed yet another ST189 isolate that was isolated in 2000 from an Australian CF patient. Altogether, these findings underscore the ubiquity of *B. multivorans* strains in different niches and on different continents.

To gain insight into the genome biology of *B. multivorans*, we analyzed all protein-coding genes in terms of homology, specificity, chromosome location and predicted function (i.e. COG category). Firstly, we identified orthologous genes because the conservation of genes may hold clues about which genes are essential for the species-specific lifestyle of *B. multivorans*. Secondly, we mapped the chromosome location of each CDS because the different chromosomes are associated with different gene copy numbers, mutation rates and expression levels and because the chromosomal location of a gene has an influence on its evolutionary course [31, 43]. Finally, we assigned each CDS to a COG to assess which biological functions were over- or underrepresented [29]. A large fraction of the orthologs (72%) was present in all nine *B. multivorans* genomes (Fig 3), showing that the *B. multivorans* isolates possessed a large set of genes regardless of their isolation source. Accordingly, Wolfgang *et al.* [44] compared clinical and environmental isolates of *Pseudomonas aeruginosa*, which is also a significant CF pathogen, and demonstrated that most strains, regardless of source, possess the basic pathogenic mechanisms necessary to cause a wide variety of human infections.

The highly conserved multireplicon genomic structure found in the present study was in agreement with the general genome architecture of Bcc organisms [45]. Since primary chromosomes contain generally more core genes [17] it was not surprising to find that C1 was enriched with core orthologs, while C2 and C3 were enriched with ST-specific orthologs (Fig 4B). These ST-specific orthologs were enriched in genes involved in defense mechanisms and secondary metabolism (Table 4), two functional categories that are generally characterized by a large degree of strain-specificity. As shown by Cooper *et al.* [31], multiple replicons allow for long-term segregation of genes by expression rates and dispensability. This way, secondary chromosomes might serve as evolutionary test beds and the ST-specific orthologs located on C2 and C3 are expected to evolve faster.

C1 was not only enriched with core orthologs but also with orthologs showing random, CF-only and ENV-only specificity (Fig 4B) and non-orthologous CDS (Fig 2B). The enrichment of C1 with random specificity orthologs may be explained by stochastic gene loss or the fact that primarily C1 suffered from unclosed assemblies (Table 2) and annotations could therefore be missing at contig ends. Nevertheless, these findings suggest that, in contrast with the overall highly conserved nature of the largest Bcc chromosome [17], C1 harbors a rather large number of non-orthologous CDS and orthologous CDS that are found only in a smaller subset (CF-only, ENV-only) of the *B. multivorans* genomes in the present study.

Because the absence or presence of specific genes may hold clues about how *B. multivorans* differs in lifestyle and epidemiology from *B. cenocepacia*, we compared the average COG profiles of these two Bcc species. In comparison with *B. cenocepacia*, the genome of *B. multivorans* was enriched in COGs involved in translation (J) and replication (L) and deprived in COGs involved in transcription (K). Since COG category K contains many transcriptional regulators, the deprivation in this category may indicate a lower adaptability of *B. multivorans* to varying environments. This different distribution in COGs involved in information storage and processing may also reflect the overall difference in genome size between these two Bcc species (Table 2 and S3 Table). Indeed, several studies [46, 47] previously demonstrated that the categories translation (J) and replication (L) showed a strong negative correlation with genome size, while transcription (K) showed a strong positive correlation with genome size. Similarly, Carlier *et al.* [48, 49] showed that the genomes of the obligate leaf nodule endosymbionts *Candidatus* Burkholderia crenata and *Candidatus* Burkholderia kirkii were smaller, enriched in COG categories J and L and deprived in COG category K when compared to free-living, facultative endophytic *Burkholderia* species. Consequently, we may expect that larger genomes require greater regulatory capacity to control their versatile metabolic capacity, as reflected by the higher number of transcriptional regulators.

Next to the differences in average COG profile related to information storage and processing, our comparison also revealed that, as compared to *B. cenocepacia*, *B. multivorans* genomes contained less COGs involved in defense mechanisms (V) (Table 5). This finding correlates with *B. multivorans* generally being less virulent than *B. cenocepacia* [50, 51]. Similarly, Bartell *et al.* [52] recently showed that *B. cenocepacia* produces a wider array of virulence factors compared to *B. multivorans*. This difference in average COG profile was also reflected by the fact that *B. cenocepacia* genomes harbored several COGs involved in resistance to antimicrobial compounds (S4 Table). *B. multivorans* on the other hand harbored COGs that encode a nitrate reductase (Table 6), which corresponds to *B. multivorans* showing nitrate reduction activity [53, 54]. Although dissimilatory nitrate reduction could enable anaerobic growth, Schwab *et al.* [54] previously showed that Bcc bacteria are incapable of anaerobic respiration and use fermentation rather than anaerobic respiration to gain energy. Altogether, the present study did not reveal any difference in the average COG profile between *B. multivorans* and *B. cenocepacia* that could explain their difference in CF epidemiology.

In this high-throughput sequencing era it is relatively straightforward to obtain draft genome sequences to study the molecular epidemiology of bacterial pathogens [55]. While short-read sequencing platforms yield draft genome assemblies at a low cost, *Burkholderia* genomes can only be fully resolved using long-read sequencing technologies such as PacBio SMRT sequencing [56, 57]. The present study provides high-quality genome assemblies for eight *B. multivorans* isolates and the final assemblies produced closed genomes for five of the eight isolates (Table 2). Although the SMRT analysis software already produced high-quality assemblies there was still a need to further polish the resulting assemblies manually (see Methods section). The circular nature of the replicons in combination with the long-read sequencing technology resulted in artificial duplications, as exemplified by the fact that the *B. multivorans* genomes initially harbored five to seven rRNA operons, while they all harbored five copies after the manual curation. The rRNA copy number is generally quite stable within a species [58] and is thus an easy quality checkpoint when evaluating the status of PacBio assemblies.

In conclusion, the present study demonstrates that the genomic structure of *B. multivorans* is highly conserved and that the ST predicts the genomic lineage. The high-quality genome assemblies provided in the present study may serve as reference genomes for future studies using transcriptomics and proteomics to try to further elucidate the epidemiology and pathogenicity of this CF pathogen.

## Supporting information

S1 Table The frequency of orthologous versus non-orthologous CDS varies among isolates. Pearson chi-square analysis testing the independence of gene conservation (orthologous vs. non-orthologous CDS) and isolate (X^2^(8) = 1829.6, p<0.001). Each cell in the contingency represents the observed frequency and standardized residual (in between brackets) and is preceded by + or − if the standardized residual is >1.96 or <-1.96, respectively, and significant at p<0.05.
(PDF)

S2 Table The frequency of orthologous versus non-orthologous CDS varies among STs. Pearson chi-square analysis testing the independence of gene conservation (orthologous vs. non-orthologous CDS) and ST (X^2^(3) = 67.3, p<0.001). Each cell in the contingency represents the observed frequency and standardized residual (in between brackets) and is preceded by + or − if the standardized residual is >1.96 or <-1.96, respectively, and significant at p<0.05.
(PDF)

S3 Table *B. cenocepacia* genomes included in the present study. CF, cystic fibrosis; ENV, environmental.
(PDF)

S4 Table *B. cenocepacia*-specific COGs. COG categories: J, translation, ribosomal structure and biogenesis; K, transcription; L, replication, recombination and repair; B, chromatin structure and dynamics; D, cell cycle control, cell division, chromosome partitioning; V, defense mechanisms; T, signal transduction mechanisms; M, cell wall/membrane/envelope biogenesis; N, cell motility; W, extracellular structures; U, intracellular trafficking, secretion, and vesicular transport; O, posttranslational modification, protein turnover, chaperones; X, mobilome: prophages, transposons; C, energy production and conversion; G, carbohydrate transport and metabolism; E, amino acid transport and metabolism; F, nucleotide transport and metabolism; H, coenzyme transport and metabolism; I, lipid transport and metabolism; P, inorganic ion transport and metabolism; Q, secondary metabolites biosynthesis, transport and catabolism; R, general function prediction only; S, function unknown.
(PDF)

S5 Table CDS with their chromosome location and COG mapping. Ortholog, orthologous CDS; Unique, non-orthologous CDS. C1, chromosome 1; C2, chromosome 2; C3, chromosome 3. TL4B, translocation from C1 to C2 in the ST650-CF isolate. COG categories: J, translation, ribosomal structure and biogenesis; K, transcription; L, replication, recombination and repair; B, chromatin structure and dynamics; D, cell cycle control, cell division, chromosome partitioning; V, defense mechanisms; T, signal transduction mechanisms; M, cell wall/membrane/envelope biogenesis; N, cell motility; W, extracellular structures; U, intracellular trafficking, secretion, and vesicular transport; O, posttranslational modification, protein turnover, chaperones; X, mobilome: prophages, transposons; C, energy production and conversion; G, carbohydrate transport and metabolism; E, amino acid transport and metabolism; F, nucleotide transport and metabolism; H, coenzyme transport and metabolism; I, lipid transport and metabolism; P, inorganic ion transport and metabolism; Q, secondary metabolites biosynthesis, transport and catabolism; R, general function prediction only; S, function unknown.
(XLSX)

S6 Table Orthologous protein families with their specificity, chromosome location and COG mapping. Core, present in all eight (spec-profile) or nine (spec-profile-atcc, including ATCC 17616) isolates; ST, specific for isolates of one or more STs; ENV-only, only occurring in environmental isolates; CF-only, only occurring in CF isolates; Random, randomly present. C1, chromosome 1; C2, chromosome 2; C3, chromosome 3. TL4B, translocation from C1 to C2 in the ST650-CF isolate. COG categories: J, translation, ribosomal structure and biogenesis; K, transcription; L, replication, recombination and repair; B, chromatin structure and dynamics; D, cell cycle control, cell division, chromosome partitioning; V, defense mechanisms; T, signal transduction mechanisms; M, cell wall/membrane/envelope biogenesis; N, cell motility; W, extracellular structures; U, intracellular trafficking, secretion, and vesicular transport; O, posttranslational modification, protein turnover, chaperones; X, mobilome: prophages, transposons; C, energy production and conversion; G, carbohydrate transport and metabolism; E, amino acid transport and metabolism; F, nucleotide transport and metabolism; H, coenzyme transport and metabolism; I, lipid transport and metabolism; P, inorganic ion transport and metabolism; Q, secondary metabolites biosynthesis, transport and catabolism; R, general function prediction only; S, function unknown.
(XLSX)

S1 Fig Parsimony tree showing the relatedness of the genomes in terms of gene content. Scale bar represents number of changes of state required in each character. The tree was rooted on the branch with the largest branch length.
(PDF)

S2 Fig Average COG profiles of *B. multivorans* and *B. cenocepacia*. Bar plot showing the average number of CDS per genome per COG category. COG categories: J, translation, ribosomal structure and biogenesis; K, transcription; L, replication, recombination and repair; B, chromatin structure and dynamics; D, cell cycle control, cell division, chromosome partitioning; V, defense mechanisms; T, signal transduction mechanisms; M, cell wall/membrane/envelope biogenesis; N, cell motility; W, extracellular structures; U, intracellular trafficking, secretion, and vesicular transport; O, posttranslational modification, protein turnover, chaperones; X, mobilome: prophages, transposons; C, energy production and conversion; G, carbohydrate transport and metabolism; E, amino acid transport and metabolism; F, nucleotide transport and metabolism; H, coenzyme transport and metabolism; I, lipid transport and metabolism; P, inorganic ion transport and metabolism; Q, secondary metabolites biosynthesis, transport and catabolism; R, general function prediction only; S, function unknown.
(PDF)

S3 Fig Multiple genome alignment in Mauve. The eight sequenced *B. multivorans* isolates from this study were aligned using Mauve [28] against *B. multivorans* strain ATCC 17616 (PRJNA17407) as a reference.
(TIF)
