# Supplementary material for: Comparative genomics of Burkholderia multivorans, a ubiquitous pathogen with a highly conserved genomic structure
Source: PLoS One. 2017 Apr 21;12(4):e0176191. doi: 10.1371/journal.pone.0176191 (PMC5400248; doi:10.1371/journal.pone.0176191)
Supplement: S1 Table — Pearson chi-square analysis testing the independence of gene conservation (orthologous vs. non-orthologous CDS) and isolate (X2(8) = 1829.6, p<0.001). Each cell in the contingency represents the observed frequency and standardized residual (in between brackets) and is preceded by + or − if the standardized residual is >1.96 or <-1.96, respectively, and significant at p<0.05. (PDF) [file pone.0176191.s001.pdf]

S1 Table. The frequency of orthologous versus non-orthologous CDS varies among isolates. Pearson chi-square analysis testing the independence of gene conservation (orthologous vs. non-orthologous CDS) and isolate ($X^2(8)=1829.6$, $p<0.001$). Each cell in the contingency represents the observed frequency and standardized residual (in between brackets) and is preceded by + or − if the standardized residual is >1.96 or <-1.96, respectively, and significant at $p<0.05$.

**Isolate**		**Orthologous CDS**		**Non-orthologous CDS**
ST180-ENV		5271 (-0.397)		523 (1.301)
ST180-CF	+	5266 (2.643)	−	285 (-8.658)
ST189-ENV	+	5144 (2.025)	−	323 (-6.632)
ST189-CF	+	5132 (2.540)	−	283 (-8.319)
ST287-ENV	+	5494 (2.589)	−	306 (-8.480)
ST287-CF		5505 (-1.668)	+	650 (5.462)
ST650-ENV	+	5275 (2.209)	−	319 (-7.235)
ST650-CF		5250 (1.795)	−	349 (-5.879)
ATCC 17616	−	4893 (-10.988)	+	1365 (35.988)
