# Supplementary material for: Comparative genomics of Burkholderia multivorans, a ubiquitous pathogen with a highly conserved genomic structure
Source: PLoS One. 2017 Apr 21;12(4):e0176191. doi: 10.1371/journal.pone.0176191 (PMC5400248; doi:10.1371/journal.pone.0176191)
Supplement: S2 Table — Pearson chi-square analysis testing the independence of gene conservation (orthologous vs. non-orthologous CDS) and ST (X2(3) = 67.3, p<0.001). Each cell in the contingency represents the observed frequency and standardized residual (in between brackets) and is preceded by + or − if the standardized residual is >1.96 or <-1.96, respectively, and significant at p<0.05. (PDF) [file pone.0176191.s002.pdf]

S2 Table. The frequency of orthologous versus non-orthologous CDS varies among STs. Pearson chi-square analysis testing the independence of gene conservation (orthologous vs. non-orthologous CDS) and ST ($X^2(3)=67.3$, $p<0.001$). Each cell in the contingency represents the observed frequency and standardized residual (in between brackets) and is preceded by + or − if the standardized residual is >1.96 or <-1.96, respectively, and significant at $p<0.05$.

ST	Orthologous CDS	Non-orthologous CDS
ST180	10537 (-0.471)	808 (1.757)
ST189	10276 (1.217)	− 606 (-4.541)
ST287	10999 (-1.473)	+ 956 (5.499)
ST650	10525 (0.797)	− 668 (-2.974)
