# Supplementary material for: Comparative genomics of Burkholderia multivorans, a ubiquitous pathogen with a highly conserved genomic structure
Source: PLoS One. 2017 Apr 21;12(4):e0176191. doi: 10.1371/journal.pone.0176191 (PMC5400248; doi:10.1371/journal.pone.0176191)
Supplement: S3 Table — CF, cystic fibrosis; ENV, environmental. (PDF) [file pone.0176191.s003.pdf]

S3 Table. ***B. cenocepacia*** **genomes included in the present study.** CF, cystic fibrosis; ENV, environmental.

Isolate	Genomovar	Project	Isolation source	Size (bp)	CDS
J2315	IIIA	PRJNA339	CF	8,055,782	7,116
H111	IIIA	PRJNA69823	CF	7,714,893	6,932
K56-2Valvano	IIIA	PRJNA62783	CF	7,750,255	6,793
AU1054	IIIB	PRJNA13919	CF	7,279,116	6,477
HI2424	IIIB	PRJNA13918	ENV	7,702,840	6,919
MC0-3	IIIB	PRJNA17929	ENV	7,971,389	7,008
