# Supplementary material for: Comparative genomics of Burkholderia multivorans, a ubiquitous pathogen with a highly conserved genomic structure
Source: PLoS One. 2017 Apr 21;12(4):e0176191. doi: 10.1371/journal.pone.0176191 (PMC5400248; doi:10.1371/journal.pone.0176191)
Supplement: S4 Table — COG categories: J, translation, ribosomal structure and biogenesis; K, transcription; L, replication, recombination and repair; B, chromatin structure and dynamics; D, cell cycle control, cell division, chromosome partitioning; V, defense mechanisms; T, signal transduction mechanisms; M, cell wall/membrane/envelope biogenesis; N, cell motility; W, extracellular structures; U, intracellular trafficking, secretion, and vesicular transport; O, posttranslational modification, protein turnover, chaperones; X, mobilome: prophages, transposons; C, energy production and conversion; G, carbohydrate transport and metabolism; E, amino acid transport and metabolism; F, nucleotide transport and metabolism; H, coenzyme transport and metabolism; I, lipid transport and metabolism; P, inorganic ion transport and metabolism; Q, secondary metabolites biosynthesis, transport and catabolism; R, general function prediction only; S, function unknown. (PDF) [file pone.0176191.s004.pdf]

S4 Table. ***B. cenocepacia*-specific COGs.** COG categories: J, translation, ribosomal structure and biogenesis; K, transcription; L, replication, recombination and repair; B, chromatin structure and dynamics; D, cell cycle control, cell division, chromosome partitioning; V, defense mechanisms; T, signal transduction mechanisms; M, cell wall/membrane/envelope biogenesis; N, cell motility; W, extracellular structures; U, intracellular trafficking, secretion, and vesicular transport; O, posttranslational modification, protein turnover, chaperones; X, mobilome: prophages, transposons; C, energy production and conversion; G, carbohydrate transport and metabolism; E, amino acid transport and metabolism; F, nucleotide transport and metabolism; H, coenzyme transport and metabolism; I, lipid transport and metabolism; P, inorganic ion transport and metabolism; Q, secondary metabolites biosynthesis, transport and catabolism; R, general function prediction only; S, function unknown.

COG	COG name	COG category
COG0063	NAD(P)H-hydrate repair enzyme Nnr, NAD(P)H-hydrate dehydratase domain	F
COG0213	Thymidine phosphorylase	F
COG0274	Deoxyribose-phosphate aldolase	F
COG0295	Cytidine deaminase	F
COG0520	Selenocysteine lyase/Cysteine desulfurase	E
COG0530	Ca2+/Na+ antiporter	P
COG0569	Trk K+ transport system, NAD-binding component	P
COG0639	Diadenosine tetraphosphatase ApaH/serine/threonine protein phosphatase, PP2A family	T
COG0738	Fucose permease	G
COG1027	Aspartate ammonia-lyase	E
COG1056	Nicotinamide mononucleotide adenylyltransferase	H
COG1090	NAD dependent epimerase/dehydratase family enzyme	R
COG1169	Isochorismate synthase EntC	HQ
COG1368	Phosphoglycerol transferase MdoB or a related enzyme of AlkP superfamily	M
COG1501	Alpha-glucosidase, glycosyl hydrolase family GH31	G
COG1510	DNA-binding transcriptional regulator GbsR, MarR family	K
COG1513	Cyanate lyase	P
COG1586	S-adenosylmethionine decarboxylase or arginine decarboxylase	E
COG1621	Sucrose-6-phosphate hydrolase SacC, GH32 family	G
COG1647	Esterase/lipase	Q
COG1708	Predicted nucleotidyltransferase	R
COG1944	Ribosomal protein S12 methylthiotransferase accessory factor YcaO	J
COG1957	Inosine-uridine nucleoside N-ribohydrolase	F
COG1972	Nucleoside permease NupC	F
COG2116	Formate/nitrite transporter FocA, FNT family	P
COG2140	Oxalate decarboxylase/archaeal phosphoglucose isomerase, cupin superfamily	G
COG2206	HD-GYP domain, c-di-GMP phosphodiesterase class II (or its inactivated variant)	T

Continued on next page

Continued from previous page

COG	COG name	COG category
COG2234	Zn-dependent amino- or carboxypeptidase, M28 family	O
COG2258	Uncharacterized conserved protein YiiM, contains MOSC domain	S
COG2509	Uncharacterized FAD-dependent dehydrogenase	R
COG2602	Beta-lactamase class D	V
COG2936	Predicted acyl esterase	R
COG2974	DNA recombination-dependent growth factor C	L
COG3011	Predicted thiol-disulfide oxidoreductase YuxK, DCC family	R
COG3147	Cell division protein DedD (periplasmic protein involved in septation)	D
COG3177	Fic family protein	K
COG3208	Surfactin synthase thioesterase subunit	Q
COG3227	Zn-dependent metalloprotease	O
COG3238	Uncharacterized membrane protein YdcZ, DUF606 family	S
COG3300	MHYT domain, NO-binding membrane sensor	T
COG3393	Predicted acetyltransferase, GNAT family	R
COG3422	Uncharacterized conserved protein YegP, UPF0339 family	S
COG3437	Response regulator c-di-GMP phosphodiesterase, RpfG family, contains REC and HD-GYP domains	T
COG3467	Nitroimidazol reductase NimA or a related FMN-containing flavoprotein, pyridoxamine 5-phosphate oxidase superfamily	V
COG3510	Cephalosporin hydroxylase	V
COG3525	N-acetyl-beta-hexosaminidase	G
COG3530	Uncharacterized conserved protein, DUF3820 family	S
COG3537	Putative alpha-1,2-mannosidase	G
COG3602	Uncharacterized protein	S
COG3637	Opacity protein and related surface antigens	M
COG3670	Carotenoid cleavage dioxygenase or a related enzyme	Q
COG3766	Uncharacterized membrane protein YjfL, UPF0719 family	S
COG3963	Phospholipid N-methyltransferase	I
COG3979	Chitodextrinase	G
COG4117	Thiosulfate reductase cytochrome b subunit	P
COG4260	Membrane protease subunit, stomatin/prohibitin family, contains C-terminal Zn-ribbon domain	O
COG4262	Predicted spermidine synthase with an N-terminal membrane domain	R
COG4285	Uncharacterized conserved protein , conains N-terminal glutamine amidotransferase (GATase1)-like domain	R
COG4448	L-asparaginase II	E
COG4452	Inner membrane protein involved in colicin E2 resistance	V
COG4551	Predicted protein tyrosine phosphatase	R
COG4589	Predicted CDP-diglyceride synthetase/phosphatidate cytidylyltransferase	R
COG4682	Uncharacterized membrane protein YiaA	S
COG4693	Oxidoreductase (NAD-binding), involved in siderophore biosynthesis	P

Continued on next page

Continued from previous page

**COG**	**COG name**	**COG category**
COG4773	Outer membrane receptor for ferric coprogen and ferric-rhodotorulic acid	P
COG4922	Predicted SnoaL-like aldol condensation-catalyzing enzyme	R
COG5002	Signal transduction histidine kinase	T
COG5460	Uncharacterized conserved protein, DUF2164 family	S
COG5472	Predicted small integral membrane protein	S
COG5500	Uncharacterized membrane protein	S
COG5528	Uncharacterized membrane protein	S
COG5612	Uncharacterized membrane protein	S
