# Supplementary material for: Comparative genomics of Burkholderia multivorans, a ubiquitous pathogen with a highly conserved genomic structure
Source: PLoS One. 2017 Apr 21;12(4):e0176191. doi: 10.1371/journal.pone.0176191 (PMC5400248; doi:10.1371/journal.pone.0176191)

S1 Fig. Parsimony tree showing the relatedness of the genomes in terms of gene content. Scale bar represents number of changes of state required in each character. The tree was rooted on the branch with the largest branch length.

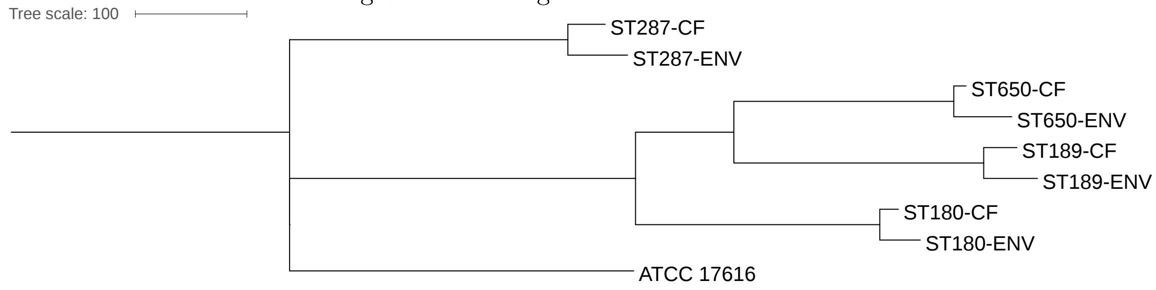

Supplement: S1 Fig — Scale bar represents number of changes of state required in each character. The tree was rooted on the branch with the largest branch length. (PDF) [file pone.0176191.s007.pdf]
