# Supplementary figures and images for: Comparative genomics of Burkholderia multivorans, a ubiquitous pathogen with a highly conserved genomic structure
Source: PLoS One. 2017 Apr 21;12(4):e0176191. doi: 10.1371/journal.pone.0176191 (PMC5400248; doi:10.1371/journal.pone.0176191)

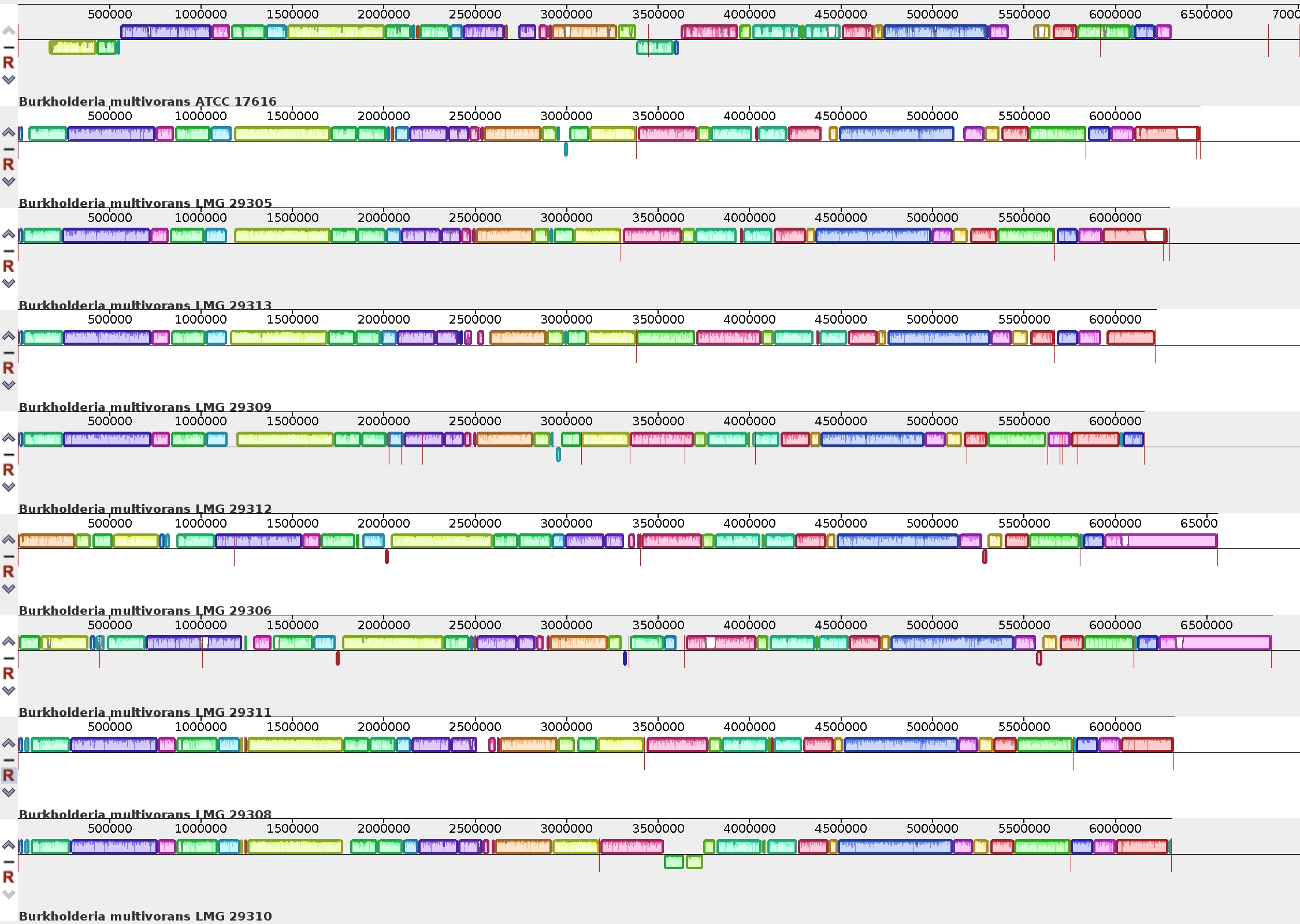

Supplement: S3 Fig — The eight sequenced B. multivorans isolates from this study were aligned using Mauve [28] against B. multivorans strain ATCC 17616 (PRJNA17407) as a reference. (TIF) [file pone.0176191.s009.tif]
